# Licensed Medical Cannabis Dispensaries: Exploring Linkages With Neighborhood Characteristics

**DOI:** 10.1093/geroni/igaf122.1967

**Published:** 2025-12-31

**Authors:** Divya Bhagianadh, Rebecca Ellis, Julie Bobitt

**Affiliations:** University of Arkansas, Fayetteville, Arkansas, United States; University of Arkansas, Fayetteville, Arkansas, United States; University of Illinois at Chicago, Chicago, Illinois, United States

## Abstract

This study explores the linkages between location of Medical Cannabis Dispensaries (MCDs) across 12 states with comprehensive medical cannabis programs and socioeconomic, and health characteristics of the neighborhood in which they are located. We mapped the location of 1,912 MCDs that were licensed to operate in January 2025 to 885 (12%) zip codes. The remaining 6,319 (88%) zip codes constitute the control group. We merged this dataset with zip code-level data on community characteristics from the social vulnerability index and county-level characteristics from the 2024 county health rankings. Multivariate logistic regression models showed that uninsured rates (OR 1.02, p-value<0.05); single parent household rates (OR 1.01, p-value<0.01); housing burden (OR 1.03, p-value<0.001); Hispanic population (OR 1.02, p-value<0.01), and being a medium metro ( vs. large central metro) (OR 1.78, p-value<0.01) were associated with higher odds of having an MCD. Households without internet (OR 0.97, p-value<0.001); being non-core or rural area (OR 0.40, p-value<0.01); fair or poor population health status (OR 0.88, p-value<0.05), and higher food environment index values (OR 0.74, p-value<0.05) were associated with lower odds of MCD. We found no association between number of physicians, drug overdose death rates, and smoking and excessive drinking rates in the area and presence of MCDs. These results contradict previous studies that found lower proportion of licensed dispensaries in locations with higher levels of socio-economic vulnerabilities. Our finding that licensed dispensaries and subsequently quality assured licensed product availability might be lower in areas with poorer health status and rural areas warrants further exploration.

